# Sensitivity and specificity of Nanopore sequencing for detecting carbapenem and 3rd-generation cephalosporin-resistant Enterobacteriaceae in urine samples: Real-time simulation with public antimicrobial resistance gene database

**DOI:** 10.1016/j.heliyon.2024.e35816

**Published:** 2024-08-05

**Authors:** Kornthara Kawang, Pannaporn Thongsuk, Pornsawan Cholsaktrakool, Songtham Anuntakarun, Pattapon Kunadirek, Natthaya Chuaypen, Sumanee Nilgate, Tanittha Chatsuwan, Intawat Nookaew, Nicha Sangpiromapichai, Voraphoj Nilaratanakul

**Affiliations:** a Division of Infectious Diseases, Department of Medicine, Faculty of Medicine, Chulalongkorn University and King Chulalongkorn Memorial Hospital, Thai Red Cross Society, Bangkok, 10330, Thailand; b Program in Bioinformatics and Computational Biology, Graduate School, Chulalongkorn University, Bangkok, 10330, Thailand; c Center of Excellence in Hepatitis and Liver Cancer, Department of Biochemistry, Faculty of Medicine, Chulalongkorn University, Bangkok, 10330, Thailand; d Metabolic Disease in Gastrointestinal and Urinary System Research Unit, Department of Biochemistry, Faculty of Medicine, Chulalongkorn University, Bangkok, 10330, Thailand; e Bumrungrad International Hospital, Bangkok, 10110, Thailand; f Department of Microbiology, Faculty of Medicine, Chulalongkorn University and King Chulalongkorn Memorial Hospital, Thai Red Cross Society, Bangkok, 10330, Thailand; g Center of Excellence in Antimicrobial Resistance and Stewardship, Faculty of Medicine, Chulalongkorn University, Bangkok, 10330, Thailand; h Department of Biomedical Informatics, University of Arkansas for Medical Sciences (UAMS), Arkansas, 72205, United States; i Master of Science Program in Medical Sciences, Faculty of Medicine, Chulalongkorn University, Bangkok, 10330, Thailand; j Healthcare-associated Infection Research Group STAR (Special Task Force for Activating Research), Chulalongkorn University, Bangkok, 10330, Thailand; k Excellence Center for Infectious Diseases, King Chulalongkorn Memorial Hospital, Thai Red Cross Society, Bangkok 10330, Thailand

**Keywords:** Nanopore, Carbapenemase, ESBL, AmpC, Enterobacteriaceae

## Abstract

**Objectives:**

To evaluate the accuracy of beta-lactamase gene detection directly from urine samples by Nanopore sequencing.

**Methods:**

DNA was extracted from bacterial pellets in spun urine. The purified DNA was then sequenced in native form by a Nanopore sequencer (MinION) to identify the organisms and beta-lactamase genes. Results were compared to routine urine cultures and standard antimicrobial susceptibility tests (AST).

**Results:**

We processed 60 urine samples of which routine cultures grew Enterobacteriaceae, including 28 carbapenem-resistant (CRE), 17 extended-spectrum beta-lactamase (ESBL) or AmpC producing, and 15 non-ESBL/AmpC phenotypes. We excluded 7 samples with extremely low DNA amounts (<1 ng/μl) for a final case of 53 in total. The sensitivity of antimicrobial resistance gene detection within 6 h, the optimal duration from real-time simulation, of Nanopore sequencing for the diagnosis of carbapenem-resistant and ceftriaxone-resistant phenotypes was 73.9 % (95%CI 56.0–91.9 %) and 81.1 % (95%CI 68.5–93.7 %), while the specificity was 96.7 % (95%CI 90.2–100.0 %) and 56.3 % (95%CI 31.9–80.6 %), respectively. The median times for MinION to generate DNA reads containing carbapenemase and ESBL/AmpC genes were 93 min (IQR 17–245.5) and 99 min (IQR 31.25–269.75) after sequencing commencement, respectively.

**Conclusions:**

Nanopore sequencing can identify bacterial genotypic resistance in urine and may enable clinicians to adjust antimicrobial therapy earlier than routine AST.

## Highlights

• Nanopore sequencing rapidly reveals antimicrobial resistance genes in urine samples.
• The accuracy of carbapenemase gene detection is acceptable (86.8 %).
• The accuracy is dependent on the quality of samples.
• Optimal antimicrobial agents can be probably adjusted as soon as the second dose.

## 1 Background

Healthcare-associated infection with multidrug-resistant bacteria (MDR) has become a major global mode of death for many hospitalized patients, regardless of their original diagnoses and underlying diseases [1]. Increased broad-spectrum antibiotic use has resulted in more MDR, while MDR has fueled greater antibiotic usage [2]. To break this cycle, an antibiotic with an optimal spectrum must be prescribed as early as possible. An empirical antibiotic initially chosen by an educated guess and administered for a few days while waiting for the result of conventional bacterial culture and antibiotic sensitivity test (AST) is often found to be an inappropriate choice. This delay increases not only MDR prevalence, but also impacts morbidity and mortality. In septic shock, every hour delay in starting effective antibiotics increases mortality by 10 % [3].

Carbapenem-resistant Enterobacteriaceae (CRE) is one of the most concerning MDRs and is associated with poor clinical outcomes [4]. Carbapenems are generally regarded as the most broad-spectrum and used as a last-resort antibiotic. Treatment options for CRE are limited, and some, like colistin, have high toxicity [5]. The prevalence of CRE has been continuously increasing [6]. Our local antibiogram from 2020 shows that 16 % of *Klebsiella pneumoniae* clinical isolates are not susceptible to carbapenems.

Extended-spectrum beta-lactamases (ESBLs) and AmpC beta-lactamases are mostly responsible for third-generation cephalosporin resistance in Enterobacteriaceae. The increase in the prevalence of ESBL/AmpC-producing bacteria has required physicians to prescribe more carbapenems [7,8], leading to their overuse and the continuous rise of CRE [2]. Rapidly and accurately classifying Enterobacteriaceae into CRE, ESBL/AmpC, and non-ESBL/AmpC groups is crucial. This could guide an improved early optimal spectrum of antibiotics, increasing the chance of better outcomes, and potentially resulting in fewer antibiotic resistance problems.

Urinary tract infections (UTIs) are mainly caused by Enterobacteriaceae and the bacterial load in urine is often sufficient for direct DNA extraction and sequencing without amplification [9]. Although some nucleic acid amplification tests are sensitive and useful, these tests, even with multiplexing, still cannot cover all types and variants of antibiotic resistance genes [10].

Nanopore sequencing technology generates real-time DNA or RNA sequences by detecting changes in electrical current as nucleic acids pass through nanopores [11]. The MinION Nanopore sequencer is portable and suitable as a point-of-care diagnostic test. To help physicians prescribe proper antibiotics for urinary tract infections as early as possible, we tested the feasibility of antibiotic resistance gene detection in urine using the Nanopore sequencer (MinION) to distinguish CRE from non-CRE cases. Speed and accuracy of carbapenemase and ESBL/AmpC gene detection were determined in this study with conventional AST as a gold standard.

## 2 Methods

### 2.1 Urine collection and storage

Adult patients whose urine was sent to the microbiology laboratory in King Chulalongkorn Memorial Hospital for routine bacterial culture and AST were consecutively enrolled after written informed consent. For minors who were too young (≤2 years old), only parental/guardian consent was obtained. All urine leftovers (up to 10 ml) from the culture were processed immediately or kept at 4^O^C for up to 8 h. The urine was centrifuged at 300g for 3 min to decrease host cell contaminates. The host-depleted supernatant was then re-centrifuged at 3000g for 10 min to form a bacterial pellet, which was kept frozen at −80^O^C.

If the urine culture result was negative or showed bacteria other than Enterobacteriaceae, the sample was excluded from the study. The AST results were classified into 3 groups: 1) CRE–nonsusceptible to any carbapenems (excluding imipenem resistance alone in *Proteus mirabilis* due to its natural resistance to this drug), 2) ESBL/AmpC–susceptible to all carbapenems, but nonsusceptible to any 3rd-generation cephalosporins, and 3) Non-ESBL/AmpC–susceptible to all carbapenems and 3rd-generation cephalosporins. The bacterial pellets were consecutively collected until reaching the calculated sample size of 28 CRE samples. Due to a higher prevalence of non-CRE (ESBL/AmpC and non-ESBL/AmpC groups), there were a lot more non-CRE samples collected. Thirty-two of them were selected for this study by random sampling.

### 2.2 DNA extraction and sequencing

DNA extraction from the frozen bacterial pellets from urine that had Enterobacteriaceae was performed using the QIAamp PowerFecal Pro DNA kit (Qiagen) according to the manufacturer's protocol. The eluted DNA was purified by AMPure XP magnetic bead (Beckman Coulter). Each DNA extract had a final volume of 50 μl, and its concentration was measured using a Qubit Fluorometer (Life Technologies). The DNA library was constructed using a Rapid Barcoding kit (SQK-RBK004, Oxford Nanopore). The library was loaded into a MinION R9 Flow Cell (12 barcodes per flow cell). The sequencing was performed on MinION Mk1B for 72 h with Software Release 20.06.4 for MinION (MinKNOW).

### 2.3 Bioinformatics analysis (Fig. 1B)

Raw fast5 files from MinION MK1B were used for demultiplexing and base-calling (high accuracy mode) with Guppy version 5.0.11 [12]. Adapter and barcode sequences were removed from the fastq files using Porechop version 0.2.4 (https://github.com/rrwick/Porechop) with the default parameters. Reads shorter than 200 nucleotides were removed using Prinseq-lite version 0.20.4 [13].

### 2.4 Identification of bacterial species and resistant genes

Reads passing Prinseq-lite criteria were aligned to the human genome (HG38) to remove host sequences. Unmapped reads were uploaded to KmerFinder 3.2 online service for species identification using the database of bacteria organisms [[14], [15], [16]]. Host-depleted reads were mapped to antibiotic-resistant genes in the ResFinder database [17] using Minimap2 [18] with default parameters. Genes encoding beta-lactamases were selected and classified into carbapenemase, ESBL, and AmpC genes according to the Beta-Lactamase DataBase (http://www.bldb.eu). Real-time simulation was done by calculating the duration between the start of Nanopore sequencing and the time stamp of the 1st read that could be mapped to carbapenemase or ESBL/AmpC genes in the ResFinder database.

### 2.5 Reference test (routine culture and AST)

Urine samples were cultured on blood agar and CLED (cysteine lactose electrolyte deficient) agar. The plates were incubated at 35 °C ± 2 °C for 24–48 h. Bacterial identification was determined by the VITEK®2XL and mass spectrometry (VITEK®MS). The antibiotic susceptibility test was determined by using the Kirby-Bauer disk diffusion method and the VITEK®2XL system. Results were interpreted according to the Clinical Laboratory Standard Institute guidelines (30th edition CLSI supplementary M100, 2020) [19].

### 2.6 Data collection

Demographic data, diagnoses, underlying diseases, and results from urine analyses, routine urine bacterial cultures, and antibiotic susceptibility test (AST) were collected from medical records and laboratory reports.

### 2.7 Statistical analysis

Sensitivity, specificity, and accuracy with 95 % confidence interval of carbapenemase or ESBL/AmpC gene detection in Enterobacteriaceae by Nanopore sequencing were calculated against the reference test of the routine AST results of phenotypic nonsusceptibility to carbapenems or 3rd-generation cephalosporins. Detection of a carbapenemase gene was considered indicative of resistance to third-generation cephalosporins, except for blaSHV-38, which hydrolyzes imipenem but has limited activity against ceftriaxone [20].

### 2.8 Sample size calculation

Assuming sensitivity and specificity of 92 %, we calculated the sample size using the formula: [$n=\frac{{Z_{\alpha /2}}^{2}pq}{d^{2}}$]. For a 95 % confidence interval with a precision of ±10 %, the approximate sample size for both CRE and non-CRE was 28 each. We increased the number of non-CRE samples, which could be acquired easier than CRE, to 32 to make all 5 MinION flow cells loaded with equal sample numbers (12 per flow cell, 60 in total) so that all results could be comparable. Non-CRE was further divided into 17 ESBL/AmpC and 15 non-ESBL/AmpC samples.

### 2.9 Antibiotic suggestion by the results of nanopore sequencing

Colistin was suggested when a carbapenemase gene was detected. The exception was *bla*_SHV-38_ without an ESBL/AmpC gene where ceftriaxone would be suggested. Carbapenem was suggested when an ESBL/AmpC gene, but not carbapenemase, was detected. Ceftriaxone was suggested when both carbapenemase and ESBL/AmpC genes were not detected.

Fluoroquinolones and aminoglycosides were excluded, since they were outside the scope of this study. Arranged from a narrower to a broader spectrum, ceftriaxone or cefixime, ceftazidime, piperacillin/tazobactam, carbapenems, and colistin were the relevant antibiotics used to treat Enterobacteriaceae in our settings. Compared with the prescribed antibiotics currently used at the time of urine culture, if Nanopore sequencing suggested the antibiotic with a broader spectrum, it was considered an escalation, while the suggestion of a narrower spectrum was considered a de-escalation (Fig. 4). From the standard AST results, an antibiotic with the narrowest spectrum that the bacteria were still susceptible to was considered the best antibiotic choice. In comparison with the spectrum of the best antibiotic choice, an antibiotic suggested by Nanopore sequencing was considered whether it was too narrow, too broad, or optimal. An antibiotic suggested by Nanopore sequencing was also considered whether it was similar to, better, or worse than the prescribed antibiotic (Supplementary Table S2).

### Role of the funding sources

There was no involvement by funding sources in the study design, specimen collection, analysis, interpretation, preparation, and submission of the manuscript.

## 3 Results

**Fig. 1:**
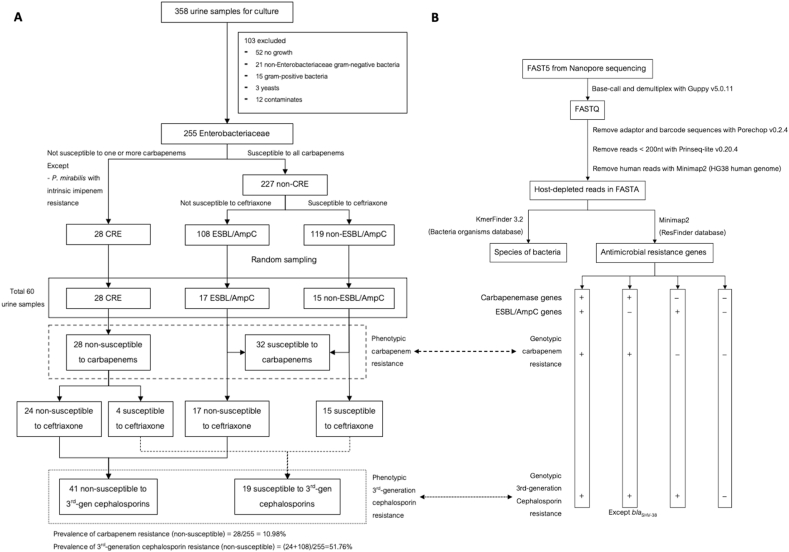
A schematic diagram of urine samples collected and utilized in this study (A) (CRE – carbapenem-resistant Enterobacteriaceae, ESBL – extended-spectrum beta-lactamase). A bioinformatic pipeline for this study (B).

From August 2020 to March 2021, 358 urine samples were collected. After exclusions and sampling, 60 urine samples from 56 patients remained for DNA extraction and sequencing (Fig. 1A). These consisted of 28 CRE, 17 ESBL/AmpC, and 15 non-ESBL/AmpC. The prevalence of carbapenem and 3rd-generation cephalosporin resistance among 255 urine samples, which were infected or colonized with Enterobacteriaceae, were 10.98 % and 51.76 %, respectively.

The demographic data of 60 urine samples are shown in Table 1. Most of the patients had underlying conditions. The most common Enterobacteriaceae found in urine was *K. pneumoniae* for the CRE group and *E. coli* for the non-CRE group. The final diagnoses were either urinary tract infection (65 %) or asymptomatic bacteriuria (35 %).

**Table 1:** Demographic and laboratory data of participants and urine samples.

Demographic and laboratory data	CRE N = 28 (percent)	ESBL/AmpC N = 17 (percent)	Non-ESBL/AmpC N = 15 (percent)	Total N = 60 (percent)
1. Sex				
Male	11(39)	6(35)	7(47)	24(40)
Female	17(61)	11(65)	8(53)	36(60)
2. Age (Median - years)	77.5	75	72.5	77
3. Underlyings				
Diabetes mellitus	12(43)	5(29)	8(53)	25(42)
Hypertension	4(14)	3(18)	2(13)	9(15)
Cerebrovascular disease	3(11)	3(18)	–	6(10)
Cancers	3(11)	1(6)	2(13)	6(10)
Chronic kidney disease	1(3.5)	4(24)	1(7)	6(10)
Neurogenic bladder	2(7)	1(6)	–	2(3)
Others	1(3.5)	1(6)	2(13)	4(7)
No underlying	2(7)	–	–	2(3)
4. Inpatient ward				
Medical	10(36)	6(35)	6(40)	22(37)
Surgical	5(17)	–	2(13)	7(12)
Pediatric	2(7)	–	–	2(3)
Outpatient	11(40)	11(65)	7(47)	29(48)
5. Diagnosis				
Urinary tract infection	15(54)	15(88)	9(60)	39(65)
Asymptomatic bacteriuria	13(46)	2(12)	6(40)	21(35)
6. Enterobacteriaceae				
*Escherichia coli*	6(21)	13(76)	10(67)	29(48)
*Klebsiella pneumoniae*	16(57)	3(18)	4(27)	23(38)
*Enterobacter* spp.	3(11)	1(6)	–	4(7)
*Klebsiella aerogenes*	2(7)	–	–	2(3)
*Proteus* spp.	–	–	1(6)	1(2)
Mixed *K. pneumoniae* and *E. coli*	1(4)	–	–	1(2)

Urine volumes ranged from 2 to 10 ml. Pyuria (WBC ≥5 cells/HPF) and significant bacteriuria (bacteria ≥ 10^5^ CFU/ml) were found in 90 % and 87 % of urine samples, respectively. The DNA amount extracted from urine bacterial pellets ranged from 0.13 to 692 ng/μl. Twenty-two samples (36.7 %) had low DNA amounts (<10 ng/μl), and seven samples (11.7 %) had very low DNA amounts (<1 ng/μl). Among 22 samples with low amounts of DNA extracts, bacterial species and antibiotic-resistant genes could not be identified by Nanopore sequencing in 3 and 6 of them, respectively. The median of the total reads per sample was 76,088 (IQR 6902–187676), while the median of the mean read length of each sample was 1591 base pairs (IQR 828–2434) (Table 2).

**Table 2:** Characteristics of sixty clinical urine samples.

Samples	Urine WBC (cells/HPF)	Colony count (cfu/ml)	DNA (ng/mcl)	Total number of reads	Mean read length (base pairs)	Non-human Reads (%)	Species identification		1st carbapenemase gene detected		1st ESBL or AmpC gene detected	
							Culture	Nanopore sequencing	Types	Time (min)^c^	Types	Time (min)^c^
1^a^	50–100	>10^5^	171	89300	2801.9	78.44	*E. cloacae* complex	*E. hormaechei*	OXA-48	15	VEB-1	10
2^a^	10–20	>10^5^	190	64895	2154.6	4.21	*E. coli*	*E. coli*	NDM-1	61	CTX-M-27	510
3^a^	10–20	>10^5^	79.6	78243	2671.9	82.71	*K. pneumoniae*	*K. pneumoniae*	–	–	CTX-M-33	10
4^a^	5–10	>10^5^	67.6	21642	2602.8	86.46	*K. pneumoniae* X2	*K. pneumoniae*	–	–	SHV-12	77
5^b^	50–100	>10^5^	179	245658	2372.6	1.52	*E. coli*	*E. coli*	–	–	CTX-M-82	869
6^b^	30–50	>10^5^	105	129680	1864.1	88.06	*E. coli*	*E. coli*	–	–	ACT-5	28
7	10–20	>10^5^	4.18	17171	562.6	10.08	*E. coli*	*E. coli*	–	–	–	–
8^a^	50–100	>10^5^	187	314766	3497.8	5.57	*E. coli* X2	*E. coli*	NDM-5	101	CTX-M-194	35
9	Numerous	>10^5^	156	73933	2271.1	18.30	*E. coli*	*E. coli*	–	–	ACT-5	378
10	50–100	>10^5^	131	69758	942.1	35.53	*E. coli*	*E. coli*	–	–	ACT-5	54
11	10–20	>10^5^	81	234022	2454.4	89.35	*P. mirabilis*	*P. mirabilis*	–	–	TEM-113	15
12	10–20	>10^5^	77.6	69672	1696	83.72	*K. pneumoniae*	*K. pneumoniae*	–	–	SHV-27	289
13^a^	10–20	>10^5^	0.174	563	190	7.28	*K. pneumoniae* X2	*K. pneumoniae*	–	–	–	–
14^a^	1–2	>10^4^	0.316	1318	320	6.15	*E. coli*	unknown	–	–	–	–
15^a^	0–1	>10^5^	3.06	32,685	3181	80.53	*E. coli*	*E. coli*	NDM-5	4	CTX-M-88	43
16^a^	N/A	>10^4^	118	43,008	4416	85.17	*K. pneumoniae* *P. aeruginosa* *E. faecalis*	*K. pneumoniae*	OXA-232	94	ACT-5	168
17^a^	30–50	10^4^–10^5^	1.71	240417	2798	4.50	*K. pneumoniae* *A. baumannii*	*K. pneumoniae* *A. baumannii*	OXA-232	13	CTX-M-218	354
18^a^	10–20	>10^5^	2.08	120491	1391	37.70	*E. cloacae* complex	*E. hormaechei* *K. pneumoniae*	OXA-232	2	ACT-16	4
19^a^	Numerous	>10^5^	42.2	80183	256	2.75	*K. pneumoniae*	*K. pneumoniae*	OXA-232	2469	–	–
20^a^	5–10	>10^5^	2.06	407580	4121	4.53	*K. pneumoniae*	*K. pneumoniae*	OXA-48	180	CTX-M-211	106
21^a^	50–100	>10^5^	2.16	94065	1242	14.02	*K. pneumoniae*	*K. pneumoniae*	OXA-232	52	CTX-M-172	140
22	30–50	>10^5^	12.2	144724	1156	3.69	*E. coli*	*E. coli*	–	–	–	–
23	5–10	>10^5^	2.26	133019	303	16.89	*E. coli*	*E. coli*	–	–	ACT-5	245
24	Numerous	>10^5^	2.52	224850	2194	8.91	*E. coli*	*E. coli*	OXA-232	93	ACT-5	196
25^b^	50–100	>10^5^	48.6	158476	1542	5.35	*E. coli*	*E. coli*	IMP-14	472	CMY-102	45
26^b^	50–100	>10^5^	7.42	403962	1253	2.20	*K. pneumoniae*	*K. pneumoniae*	–	–	CTX-M-176	75
27^b^	50–100	>10^5^	10	60341	3405	89.78	*E. coli*	*E. coli*	–	–	VEB-3	3
28^b^	50–100	>10^5^	0.324	1788	695	24.44	*E. coli*	*E. coli*	–	–	TEM-198	233
29^b^	10–20	>10^5^	54.4	201053	861	7.37	*E. coli* *C. koseri*	*E. coli*	–	–	–	–
30^b^	Numerous	>10^5^	65.4	92097	531	7.35	*E. coli* *P. aeruginosa*	*E. coli*	–	–	CTX-M-157	195
31^b^	50–100	>10^5^	23.6	64433	983	11.59	*E. coli*	*E. coli*	–	–	CTX-M-174	251
32^b^	10–20	>10^5^	5.74	52521	235	11.24	*E. cloacae*	*E. cloacae*	–	–	ACT-9	14
33^b^	30–50	>10^5^	46	276356	912	4.53	*E. coli* X2	*E. coli*	–	–	ACT-5	79
34	20–30	>10^5^	27.6	244076	1290	3.84	*E. coli*	*E. coli*	–	–	TEM-47	212
35	30–50	>10^5^	18.8	239685	692	11.92	*K. pneumoniae*	*K. pneumoniae*	–	–	–	–
36^a^	10–20	>10^5^	9.74	130116	906	59.48	*K. pneumoniae*	*K. pneumoniae*	SHV-38	224	–	–
37^a^	1–2	>10^5^	5.44	6710	817	21.58	*K. pneumoniae*	*K. pneumoniae*	OXA-232	53	CTX-M-189	677
38^a^	0–1	10^4^–10^5^	0.13	5123	322	5.68	*K. aerogenes*	*K. aerogenes*	NDM-1	531	–	–
39^a^	20–30	>10^5^	108	170793	1678	4.34	*K. pneumoniae*	*K. pneumoniae*	NDM-1	267	CTX-M-188	139
40^a^	N/A	>10^5^	494	68002	3194	23.93	*E. coli* *K. pneumoniae*	*E. coli* *K. pneumoniae*	OXA-232	159	CTX-M-27	92
41^a^	Numerous	10^4^–10^5^	0.17	3206	409	9.08	*K. aerogenes*	*K. pneumoniae*	–	–	–	–
42^a^	Numerous	10^4^–10^5^	137	193304	3334	16.15	*K. pneumoniae*	*K. pneumoniae*	–	–	DHA-1	68
43^a^	20–30	10^4^–10^5^	0.17	5162	386	3.49	*K. pneumoniae*	unknown	–	–	–	–
44^a^	30–50	>10^5^	206	136643	1120	7.49	*E. coli*	*E. coli*	NDM-6	12	–	–
45^a^	5–10	>10^5^	145	118645	1604	5.15	*K. pneumoniae*	*K. pneumoniae*	OXA-48	364	SHV-100	13
46^a^	N/A	10^4^–10^5^	9.74	132493	790	3.31	*K. pneumoniae*	*K. pneumoniae*	NDM-16	3736	–	–
47^a^	30–50	>10^5^	228	321433	2089	35.88	*E. coli*	*E. coli*	NDM-20	19	CTX-M-183	14
48^a^	N/A	>10^5^	97.8	278431	3274	6.10	*E. cloacae* complex	*E. cloacae* complex	NDM-24	92	CTX-M-15	14
49^a^	5–10	>10^5^	28.8	262069	2224	25.09	*K. pneumoniae*	*K. pneumoniae*	NDM-1	0.03	CTX-M-216	30
50^b^	Numerous	>10^5^	96.2	7478	1534	85.00	*E. coli*	*E. coli*	–	–	CTX-M-202	53
51^b^	20–30	>10^5^	2.46	2785	1821	85.60	*K. pneumoniae*	*K. pneumoniae*	–	–	–	–
52^b^	N/A	>10^5^	117	2095	2230	79.76	*E. coli*	*E. coli*	–	–	DHA-1	540
53^b^	30–50	>10^5^	159	3908	1822	75.61	*E. coli*	*E. coli*	–	–	OXA-534	3135
54^b^	20–30	>10^5^	161	5342	1799	87.89	*E. coli*	*E. coli*	–	–	CTX-M-182	312
55^b^	30–50	>10^5^	34.2	5723	2473	86.35	*K. pneumoniae*	*K. pneumoniae*	–	–	CTX-M-210	276
56	N/A	>10^5^	0.392	224	528	42.41	*E. coli*	*E. coli*	–	–	–	–
57	50–100	>10^5^	2.06	786	1579	70.87	*K. pneumoniae*	unknown	–	–	–	–
58	N/A	>10^5^	94.4	6298	1758	65.26	*E. coli*	*E. coli*	–	–	TEM-144	1015
59	30–50	>10^5^	111	11464	1199	4.56	*K. pneumoniae*	*K. pneumoniae*	–	–	–	–
60	Numerous	>10^5^	692	8131	3567	7.19	*E. coli*	*E. coli*	–	–	–	–

N/A – not available (Physicians ordered only urine culture, not urinalysis.).

X2 in species identification means 2 isolates of the same species.

a Urine samples with carbapenem-resistant Enterobacteriaceae (CRE phenotype).

b Urine samples with 3rd-generation cephalosporin-resistant Enterobacteriaceae (susceptible to all carbapenems—ESBL/AmpC phenotype).

c Calculated from the starting time of Nanopore sequencing to the time stamps of the 1st read that contained a carbapenemase or an ESBL/AmpC gene.

Bacterial species in urine samples were identified by the top matching score in KmerFinder 3.2. The species identified by Nanopore sequencing were mostly confirmed by routine bacterial culture results, except for three samples (urines 14, 43, 57), where the DNA amount was too low for any matching. In some urine samples (urines 16, 29, 30) with mixed organisms, only one organism was confirmed by Nanopore sequencing. One discordance, *K. pneumoniae* by Nanopore sequencing versus *K. aerogenes* by routine culture, was found in urine 41, of which DNA amount was also very low (Table 2).

### 3.1 Detection of beta-lactamase genes

The comparisons between bacterial genotypic and phenotypic antimicrobial resistance of all 60 urine samples are shown in Supplementary Table S1. When comparing carbapenemase gene detection by Nanopore sequencing with any carbapenem resistance detected by routine AST, the sensitivity and specificity were 75.0 % (95%CI 59.0–91.0 %) and 93.8 % (95%CI 85.4–100.0 %), respectively. The sensitivity and specificity of ESBL/AmpC/carbapenemase (except *bla*_SHV-38_) genes detection by Nanopore sequencing against 3rd-generation cephalosporin resistance by routine AST were 87.8 % (95%CI 77.8–97.8 %) and 47.4 % (95%CI 24.9–69.8 %).

Excluding samples with very low DNA amounts (<1 ng/μl) improved accuracy. Real-time simulation of Nanopore sequencing showed that the accuracy of carbapenemase and ESBL/AmpC genes detection increased until almost reached plateau at 6–7 h of Nanopore sequencing. At 6 h, excluding 7 samples with very low DNA amount (<1 ng/μl), the sensitivity and specificity for the detection of carbapenem resistance were 73.9 % (95%CI 56.0–91.9 %) and 96.7 % (95%CI 90.2–100.0 %), respectively, while the sensitivity and specificity for the detection of 3rd-generation cephalosporin resistance were 81.1 % (95%CI 68.5–93.7 %) and 56.3 % (95%CI 31.9–80.6 %). With the same conditions (6 h, DNA ≥1ng/μl), the agreements (Cohen's kappa) between genotype (Nanopore sequencing) and phenotype (AST) were substantial (0.724, 95%CI 0.534–0.915) and fair (0.373, 95%CI 0.092–0.655) for carbapenem and 3rd-generation cephalosporin resistance, respectively.

**Fig. 2:**
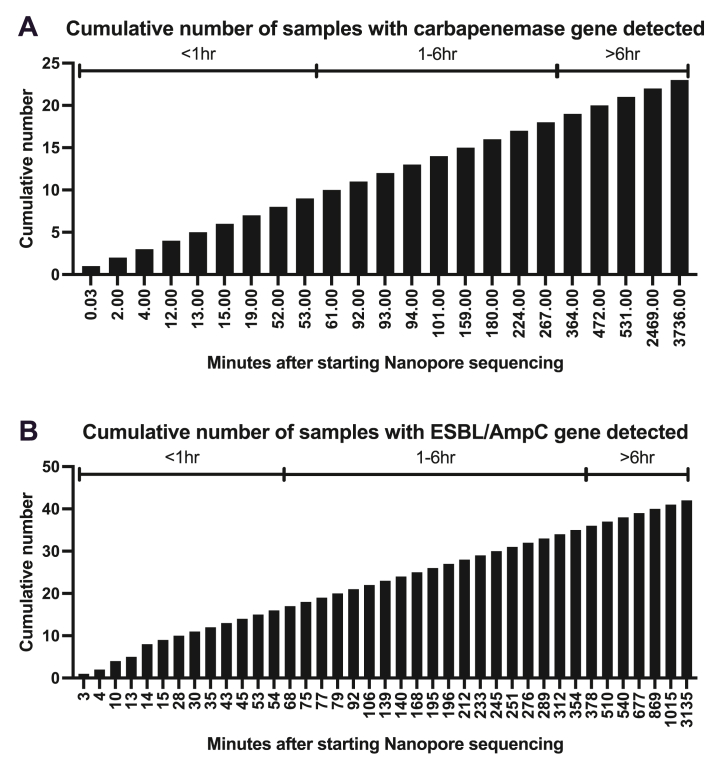
Cumulative number of urine samples of which carbapenemase (A) or ESBL/AmpC (B) gene-containing sequence reads in fast5 format were generated overtime by MinION flow cells.

The carbapenemase genes detected were *bla*_NDM-1,2,3,4,5,6,9,14,15,16,17,20,21,22,24_, *bla*_OXA-48,232_, and *bla*_SHV-38_. The ESBL/AmpC genes detected were the variants of *bla*_TEM_, *bla*_SHV_, *bla*_CTX-M_, *bla*_VEB_, *bla*_OXA_, *bla*_ACT_, *bla*_DHA_, *bla*_MIR_, and *bla*_CMY_. The time from the start of Nanopore sequencing until the generation of the 1st read in fast5 format, which would be later base-called and matched to a carbapenemase and ESBL/AmpC genes in the ResFinder database, ranged widely from 2 s to more than 2 days (Table 2 and Fig. 2) with a median of 93 min (IQR 17–245.5) for carbapenemase genes and 99 min (IQR 31.25–269.75) for ESBL/AmpC genes.

**Fig. 3:**
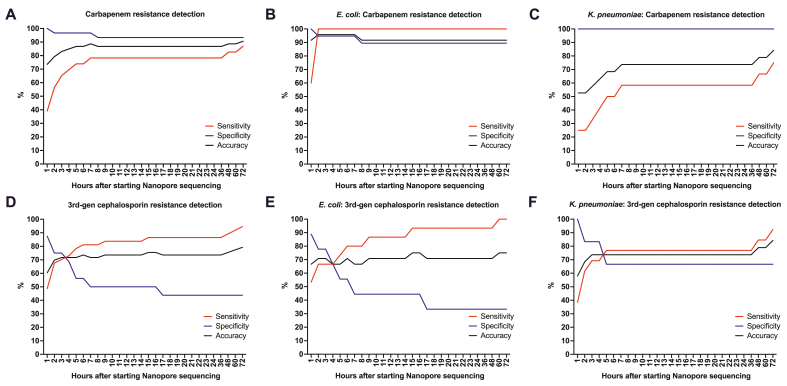
Real-time simulation of sensitivity, specificity, and accuracy of carbapenem (A, B, and C) and 3rd-generation cephalosporin (D, E, and F) resistance, predicted by Nanopore sequencing, over time after starting the sequencing in all >1 ng/μl urine samples (n = 53, A and D), *E. coli* only samples (n = 24, B and E), and *K. pneumoniae* only samples (n = 19, C and F).

The overall sensitivity, specificity, positive predictive value (PPV), negative predictive value (NPV), accuracy, and Cohen's kappa are summarized in Table 3. The effects of DNA amount, bacterial species, and total Nanopore sequencing duration on these values are also shown in Table 3 and Fig. 3.

**Table 3:**
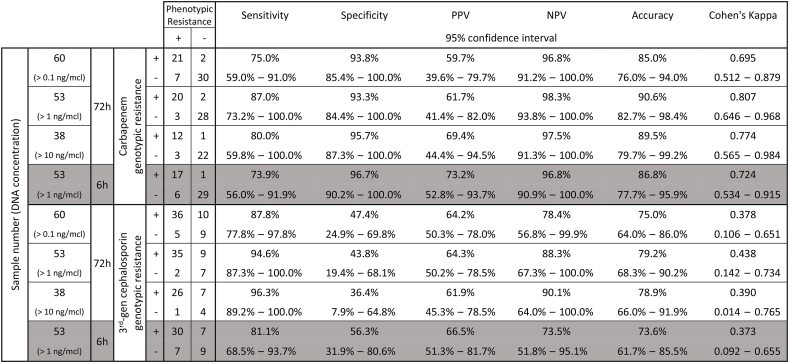
Performance of Nanopore sequencing in comparison to conventional antimicrobial susceptibility test.

- ^a^ Prevalence of urine culture positive for carbapenem- (28/255 = 10.98 %) and 3rd-generation cephalosporin- ((24 + 108)/255 = 51.76 %) resistant Enterobacteriaceae are used to calculate PPV and NPV.

- Total number of urine culture positive for Enterobacteriaceae during study period is 255.

- Total number of urine culture positive for carbapenem-resistant Enterobacteriaceae during study period is 28. Out of this, 24 are resistant to 3rd-generation cephalosporin.

- Total number of urine culture positive for Enterobacteriaceae with ESBL/AmpC phenotype during study period is 108.

**Fig. 4:**
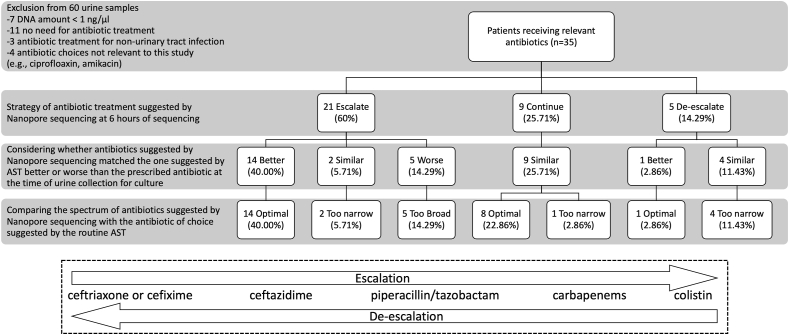
Comparison between the prescribed antibiotics, the antibiotic suggested by the results of Nanopore sequencing, and the antibiotic suggested by the antimicrobial susceptibility test. The lower panel shows the relevant antibiotics and how to define escalation and de-escalation of antibiotics in this study.

### 3.2 Antibiotic suggestion by the results of nanopore sequencing

Among 53 episodes of urine culture with urine DNA amount above 1 ng/μl, 35 of them had relevant antibiotic treatments (Fig. 4). Only 11 (31.43 %) of these actual antibiotic treatments were optimal (21 too narrow and 3 too broad), while 23 (65.71 %) of the antibiotics suggested by the Nanopore sequencing at 6 h of sequencing, were optimal (7 too narrow and 5 too broad). The antibiotics suggested by Nanopore sequencing are also matched to the antibiotics suggested by AST somewhat better than are the prescribed antibiotics—15 better, 15 similar, and 5 worse (Fig. 4 and Supplementary Table S2).

## 4 Discussion

Schmidt et al. (2017) previously reported promising results of Nanopore sequencing for detecting bacterial pathogens and antimicrobial resistance in a small sample of clinical urines and healthy urines spiked with MDR *E. coli* [9]. Our study confirmed the feasibility of this approach in a larger number of clinical urines and a wider variety of urine pathogens. This study represented real-world practice where poor-quality samples are unavoidable, leading to false negative results. Even under these conditions, this approach still performed quite well for CRE detection. Furthermore, Excluding samples with extremely low DNA amounts (<1 ng/μl) would have improved accuracy (Table 3). The results of *E. coli* and *K. pneumoniae* subgroup showed excellent sensitivity and specificity, respectively (Fig. 3), but the sample size is too small to draw a strong conclusion.

The species with the highest prevalence in the CRE group (n = 28) was *K. pneumoniae* (57 %). Twenty-three carbapenemase genes were detected by Nanopore sequencing. The most common was *bla*_OXA_ (48 %), followed by *bla*_NDM_ (39 %). This type of approach could guide antibiotic selection, since bacteria with OXA-48 (and its derivatives), not NDMs, are still susceptible to ceftazidime/avibactam, and colistin can be spared [21]. The presence and/or absence of other beta-lactamase genes could help determine whether carbapenems, 3rd-generation cephalosporins, or ampicillin should be given.

There are several advantages of real-time Nanopore sequencing over routine bacterial culture and AST. Including the 1 h spent on DNA extraction, 6 h on Nanopore sequencing, and 1 h for bioinformatic analysis, the whole process of resistance gene detection can be within 8 h, much faster than routine AST (48–72 h), making it possible to adjust the antibiotics early on. In this study, we barcoded 12 samples per MinION flow cell, which could also compromise the speed of detection. If we had sequenced only one sample per run of the MinION flow cell, the speed of detection would have likely been greatly improved, at the cost of higher price, due to the presence of 12 times more available pores per sample. The sequencing also provides additional details on species and strains which are invaluable for epidemiological studies. In this study, 2 isolates of *Enterobacter hormaechei*, a member of *Enterobacter cloacae* complex, were identified only with Nanopore sequencing.

For this study, the detection of AmpC-encoding genes was interpreted as resistance to 3rd-generation cephalosporins. However, some of them were false positive. Third-generation cephalosporin resistance is sometimes caused by overexpression of inducible AmpC-encoding genes [22]; therefore, a mere presence of these genes does not guarantee the resistance.

Some CRE might not have putative carbapenemase genes, leading to false-negative results. Porin deficiency, usually found together with ESBL or AmpC beta-lactamases, can be responsible for the carbapenem-resistant phenotype in non-carbapenemase-producing *K. pneumoniae* [23]. In our ongoing studies, we have performed the whole genome assembly of carbapenem-resistant *K. pneumoniae* in clinical isolates from our hospital and indeed found no carbapenemase-encoding gene in some isolates (data not shown).

The high error rate of Nanopore sequencing can complicate the results, especially on the ESBL genes. For instance, accumulation of some mutations can transform ampicillinase genes like *bla*_TEM-1_ or *bla*_SHV-1_ into their derivatives (e.g., *bla*_TEM-3_ or *bla*_SHV-2_), of which encoded betalactamases have an extended spectrum (a.k.a. ESBL) and can degrade both ampicillin and 3rd-generation cephalosporins [24]. Unlike species and carbapenemase gene detection which are affected by nanopore sequencing errors only slightly, errors found on ampicillinase or ESBL genes may cause misinterpretation of these genes as each other, resulting in either false positives or false negatives. Therefore, the accuracy of CRE prediction is much better than ESBL/AmpC prediction. Whole bacterial genome assembly to achieve consensus sequences might partially clean up these errors, leading to higher accuracy at the cost of longer processing time and a more complicated pipeline for real-time analysis. Hybrid assembly with Nanopore long-read sequencing and Illumina short-read sequencing can improve the assembly quality [25], but may be not practical for clinical laboratory service. Also, many samples had scanty copy number of resistant genes, not enough to make a correct consensus.

Without amplification steps, sequencing largely depends on sample quality. In real situations like our study, urine samples are frequently poor in quality. Antibiotics are commonly administered long before urine collection, significantly reducing bacterial load in urine. Low urine volume and host cell contamination can also lead to a low ratio of bacterial DNA to host DNA. This can be improved by the recently developed technique called “adaptive sampling” which can remove host DNA from the pore during the Nanopore sequencing [26]. Flora contaminations may further complicate the interpretations.

We chose to extract DNA with a bead-beating method to shorten the time needed for extraction. As a result, DNA fragments are relatively short (Table 2) and may compromise the quality of nanopore sequencing, which is more suitable with long reads. In contrast, DNA extraction methods that are specialized for long reads are relatively slow. The accuracy can be improved, but the speed needed for adjusting the antibiotics may not be achieved.

Another limitation is that about half of the samples used in this study contained CRE, which were likely to require antibiotic escalation. However, the actual prevalence of CRE in the period of this study was only 10.98 %. Thus, the usefulness of carbapenem resistance predicted by Nanopore sequencing is overinflated. Also, for non-complicated UTI, antibiotic escalation may not be always needed for the non-susceptible bacteria due to very high concentration of antibiotics in urine [27].

In Fig. 4, there were 5 cases where Nanopore sequencing suggested worse antibiotic choices than the prescribed antibiotics. Interestingly, all those worse choices were too broad. This was probably more favorable than too narrow, where patients might succumb to the antimicrobial-resistant pathogens.

The cost of Nanopore sequencing depends on the types of flow cells and multiplexing—up to 24 and 96 barcodes in MinION and PromethION flow cells, respectively. This can be from $25 up to $90 per sample, excluding equipment and bioinformatic analysis.

In conclusion, our study demonstrates the accuracy of beta-lactamase gene detection by Nanopore sequencing. To achieve the greatest potential use of Nanopore sequencing, an analysis should be attempted in real time. If the bacterial load in urine is high enough, the proper antibiotics can be suggested as early as a few minutes after sequencing. The accuracy can be further improved with the development of the new flow cell (R10.4 and beyond) which generates better quality DNA reads [28].

## Funding sources

This research was funded by Thailand Science research and Innovation Fund 10.13039/501100002873Chulalongkorn University (HEAF67300012), 10.13039/501100004704National Research Council of Thailand (10.13039/501100004704NRCT) and 10.13039/501100002873Chulalongkorn University (N42A660522), and Quality Improvement Fund, 10.13039/100007693King Chulalongkorn Memorial Hospital, the Thai Red Cross Society (HA-67-3300-C1-052). Voraphoj Nilaratanakul was supported by the Ratchadaphiseksomphot Fund, 10.13039/501100002873Chulalongkorn University (CU_GR_63_127_30_34), Grant for Development of New Faculty Staff (DNS 64_002_30_001_2 and DNS 66_007_30_001_3) Ratchadaphiseksomphot Fund, 10.13039/501100002873Chulalongkorn University, and the Ratchadapiseksompotch Fund, 10.13039/501100004776Faculty of Medicine, Chulalongkorn University (RA-MF-51/64). Kornthara Kawang was supported by Ratchadapisek Somphot Fund for Postdoctoral Fellowship, 10.13039/501100002873Chulalongkorn University. Pornsawan Cholsaktrakool was supported by 60/40 Support for Tuition Fee, 10.13039/501100002873Chulalongkorn University. Songtham Anuntakarun was supported by the Ratchadapisek Somphot Fund for Postdoctoral Fellowship, 10.13039/501100002873Chulalongkorn University. Nicha Sangpiromapichai was supported by 10.13039/501100023924CU Graduate School Thesis Grant, 10.13039/501100002873Chulalongkorn University (GCUGR1225642061M). Intawat Nookaew was partially supported by National Institute of Health (P20GM125503).

## Ethical approval statement

In accordance with the Code of Ethics of the World Medical Association (Declaration of Helsinki), this study was approved by the Institutional Review Board, Faculty of Medicine, Chulalongkorn University (IRB number 511/63; date of approval—August 4, 2020, COA number 960/2020; date of first extension—August 4, 2021, COA number 1069/2021) and the Institutional Biosafety Committee, Faculty of Medicine, Chulalongkorn University (MDCU-IBC number 016/2020). This study was also registered in the Thai Clinical Trial Registry (TCTR20200707002).

## Data sharing

All raw sequence data in fastq format have been deposited at BioProject (http://www.ncbi.nlm.nih.gov/bioproject/1123902) with accession numbers PRJNA1123902.

## CRediT authorship contribution statement

**Kornthara Kawang:** Writing – review & editing, Writing – original draft, Investigation. **Pannaporn Thongsuk:** Writing – review & editing, Writing – original draft, Investigation, Data curation. **Pornsawan Cholsaktrakool:** Writing – review & editing, Writing – original draft, Formal analysis, Data curation. **Songtham Anuntakarun:** Writing – original draft, Formal analysis. **Pattapon Kunadirek:** Writing – original draft, Formal analysis. **Natthaya Chuaypen:** Writing – original draft, Formal analysis. **Sumanee Nilgate:** Writing – original draft, Resources. **Tanittha Chatsuwan:** Writing – review & editing, Writing – original draft, Resources. **Intawat Nookaew:** Writing – original draft, Formal analysis. **Nicha Sangpiromapichai:** Writing – original draft, Investigation. **Voraphoj Nilaratanakul:** Writing – review & editing, Writing – original draft, Visualization, Supervision, Methodology, Funding acquisition, Formal analysis, Data curation, Conceptualization.

## Declaration of generative AI and AI-assisted technologies in the writing process

During the preparation of this work the author used ChatGPT 4o in order to correct grammatical mistakes. After using this tool, the author reviewed and edited the content as needed and takes full responsibility for the content of the publication.

## Declaration of competing interest

The authors declare that they have no known competing financial interests or personal relationships that could have appeared to influence the work reported in this paper.
