# Supplementary material for: Sensitivity and specificity of Nanopore sequencing for detecting carbapenem and 3rd-generation cephalosporin-resistant Enterobacteriaceae in urine samples: Real-time simulation with public antimicrobial resistance gene database
Source: Heliyon. 2024 Aug 5;10(16):e35816. doi: 10.1016/j.heliyon.2024.e35816 (PMC11382077; doi:10.1016/j.heliyon.2024.e35816)
Supplement: Multimedia component 1 [file mmc1.docx]

**Supplementary Appendix**

**Table S1.** Comparison between discovery of betalactamase genes from MinION Nanopore sequencing (genotypic resistance) and routine culture with standard antimicrobial susceptibility test against beta-lactam antibiotics (phenotypic resistance) of all 60 urine samples

|  | Urine1* | | Urine2* | | Urine3* | | Urine4* | |
| --- | --- | --- | --- | --- | --- | --- | --- | --- |
|  | MinION | Culture | MinION | Culture | MinION | Culture | MinION | Culture |
| Bacteria | *Enterobacter hormaechei* | *Enterobacter*  *cloacae* complex | *E. coli* | *E. coli* | *K. pneumoniae* | *K. pneumoniae* | *K. pneumoniae* | *K. pneumoniae*  2 isolates |
| Ampicillin | *bla*_TEM-1B_ | - |  | R | *bla*_TEM-1C,1B,1D_ | R |  | R/R |
| Amoxicillin/  Clav |  | R |  | R |  | R |  | R/I |
| Piperacillin/  Taz |  | I |  | R |  | R |  | R/R |
| Cefazolin |  | R |  | R |  | R |  | R/R |
| Cefuroxime |  | R |  | R |  | R |  | R/R |
| Ceftriaxone | *bla*_ACT-7,15_  *bla*_OXA-1,10,14,17,233,240,244,246,251,252,256,320,368,439,454,505,515,519,520,534,547_  *bla*_VEB-1,4,5,7_  *bla*_DHA-1,6,7,14,15,27_  *bla*_TEM_*_-_*_34,36,70,95,101,104,143,166,176,198,206,207,214,215,220,231,233_ | R | *bla*_CTX-M-27_ | R | *bla*_CTX-M-15,33,157,172,176,182,189,193,204,207,208_  *bla*_SHV-100_ | R | *bla*_SHV-11,12_ | R/R |
| Cefepime |  | SDD |  | R |  | R |  | SDD/SDD |
| Doripenem |  | S |  | R |  | R |  | I/I |
| Ertapenem |  | S |  | - |  | - |  | S/S |
| Imipenem | *bla*_OXA-48_ | I |  | R |  | R |  | I/I |
| Meropenem |  | S | *bla*_NDM-1,6,16_ | R |  | R |  | S/S |

|  | Urine5** | | Urine6** | | Urine7 | | Urine8**/* | |
| --- | --- | --- | --- | --- | --- | --- | --- | --- |
|  | MinION | Culture | MinION | Culture | MinION | Culture | MinION | Culture |
| Bacteria | *E. coli* | *E. coli* | *E. coli* | *E. coli* | *E. coli* | *E. coli* | *E. coli* | *E. coli*  2 isolates |
| Ampicillin | *bla*_TEM-1B_ | R |  | R |  | R | *bla*_OXA-1_  *, bla*_TEM-1B_ | R/R |
| Amoxicillin/Clav |  | R |  | S |  | R |  | I/R |
| Piperacillin/Taz |  | S |  | S |  | R |  | S/R |
| Cefazolin |  | R |  | R |  | S |  | R/R |
| Cefuroxime |  | R |  | R |  | I |  | R/R |
| Ceftriaxone | *bla*_CTX-M-82_ | R | *bla*_CTX-M-15,103,117,156,180_  *bla*_OXA-10_ | R |  | S | *bla*_CMY-42_*,*  *bla*_CTX-M-15,114,127,183,188-9_ | R/R |
| Cefepime |  | SDD |  | SDD |  | S |  | SDD/R |
| Doripenem |  | S |  | S |  | S |  | S/I |
| Ertapenem |  | S |  | S |  | S |  | S/R |
| Imipenem |  | S |  | S |  | S |  | S/R |
| Meropenem |  | S |  | S |  | S | *bla*_NDM-17,20,_ *bla*_NDM-4,5_ | S/R |

|  | Urine9 | | Urine10 | | Urine11 | | Urine12 | |
| --- | --- | --- | --- | --- | --- | --- | --- | --- |
|  | MinION | Culture | MinION | Culture | MinION | Culture | MinION | Culture |
| Bacteria | *E. coli* | *E. coli* | *E. coli* | *E. coli* | *Proteus mirabilis* | *Proteus mirabilis* | *K. pneumoniae* | *K. pneumoniae* |
| Ampicillin | *bla*_TEM-1B_ | S | *bla*_TEM-1B_ | R | *bla*_OXA-1,_ *bla*_TEM-1B,1c_ | R |  | R |
| Amoxicillin/  Clav |  | S |  | I |  |  |  | S |
| Piperacillin/  Taz |  | S |  | I |  | S |  | S |
| Cefazolin |  | S |  | S |  | S |  | S |
| Cefuroxime |  | S |  | S |  | S |  | S |
| Ceftriaxone |  | S | *bla*_TEM-105_ | S | *bla*_OXA-320,534,_  *bla*_TEM-15,28-30,33-34,55,57,70,76,79,95,104-105,122,127,143,148,156,164,166,176,186,196,198.201,206-9,214-6,220,230,234_ | S | *bla*_SHV-27_ | S |
| Cefepime |  | S |  | S |  | S |  | S |
| Doripenem |  | S |  | S |  | - |  | S |
| Ertapenem |  | S |  | S |  | S |  | S |
| Imipenem |  | S |  | S |  | R |  | S |
| Meropenem |  | S |  | S |  | S |  | S |

|  | Urine 13*/** | | Urine 14* | | Urine 15* | | Urine 16* | |
| --- | --- | --- | --- | --- | --- | --- | --- | --- |
|  | MinION | Culture | MinION | Culture | MinION | Culture | MinION | Culture |
| Bacteria | *K.*  *pneumoniae* | *K.*  *pneumoniae* | - | *E. coli* | *E. coli* | *E. coli* | *K. pneumoniae* | *K. pneumoniae/*  *P. aeruginosa/*  *E. faecium* |
| Ampicillin |  | R/R |  | R | *bla*_TEM-1B_ | R | *bla*_TEM 1B_ | R/-/R |
| Amoxicillin/ Clav |  | R/R |  | R |  | R |  | R/-/- |
| Piperacillin/ Taz |  | R/R |  | R |  | R |  | R/S/- |
| Cefazolin |  | R/R |  | R |  | R |  | R/-/- |
| Cefuroxime |  | R/R |  | R |  | R |  | R/-/- |
| Ceftriaxone |  | R/R |  | R | *bla*_CTX-M-55,88,104,117,172,173,189,193,204,209_  *bla*_CMY-2,32,62,121,137_  *bla* _ACT-5_  *bla* _MIR-5_  *bla*_OXA-1,47,224,534_  *bla* _TEM 30,33,34,70,52B,105,176,216,226_ | R | *bla* _TEM 30,57,196,215_  *bla* _ACT-5,14_ | R/-/- |
| Cefepime |  | R/R |  | R |  | R |  | R/-/- |
| Doripenem |  | I/S |  | R |  | R |  | R/I/- |
| Ertapenem |  | - |  | - |  | - |  | - |
| Imipenem |  | I/S |  | R | *bla*_NDM-1,5,14,20,17_ | R |  | R/R/- |
| Meropenem |  | S/S |  | R | *bla*_OXA-232_ | R | *bla*_OXA-232_ | R/R/- |

|  | Urine 17* | | Urine 18* | | Urine 19* | | Urine 20* | |
| --- | --- | --- | --- | --- | --- | --- | --- | --- |
|  | MinION | Culture | MinION | Culture | MinION | Culture | MinION | Culture |
| Bacteria | *K. pneumoniae*/  *A. baumannii* | *K. pneumoniae/*  *A. baumannii* | *Enterobacter hormaechei/*  *K. pneumoniae* | *Enterobacter cloacae* complex | *K. pneumoniae* | *K. pneumoniae* | *K. pneumoniae* | *K. pneumoniae* |
| Ampicillin | *bla*_TEM-1A_ | R/- |  | - |  | R | *bla*_TEM 1A,1C_ | R |
| Amoxicillin/Clav |  | R/- |  | R |  | R |  | R |
| Piperacillin/Taz |  | R/R |  | R |  | R |  | R |
| Cefazolin |  | R/- |  | R |  | R |  | R |
| Cefuroxime |  | R/- |  | R |  | R |  | R |
| Ceftriaxone | *bla*_OXA-1,9,181,484_  *bla*_CTX-M-15,117,184,189,209,218_  *bla*_SHV-11,148_ | R/R | *bla* _ACT-16_ | R |  | R | *bla*_TEM 40,55_  *bla* _OXA-244_  *bla*_SHV-98_  *bla*_CTX-M-15,150,211_ | R |
| Cefepime |  | R/- |  | S |  | R |  | R |
| Doripenem |  | R/R |  | R |  | R |  | R |
| Ertapenem |  |  |  | - |  | - |  | - |
| Imipenem |  | R/R |  | R |  | R |  | I |
| Meropenem | *bla*_OXA-232_ | R/R | *bla*_OXA-232_ | R | *bla*_OXA-232_ | R | *bla*_OXA-48_ | R |

|  | Urine 21* | | Urine 22 | | Urine 23 | | Urine 24 | |
| --- | --- | --- | --- | --- | --- | --- | --- | --- |
|  | MinION | Culture | MinION | Culture | MinION | Culture | MinION | Culture |
| Bacteria | *K. pneumoniae* | *K. pneumoniae* | *E. coli* | *E. coli* | *E. coli* | *E. coli* | *E. coli* | *E. coli* |
| Ampicillin | *bla*_TEM-1A, 1C_ | R |  | - |  | S |  | R |
| Amoxicillin/  Clav |  | R |  | S |  | S |  | I |
| Piperacillin/Taz |  | R |  | S |  | S |  | S |
| Cefazolin |  | R |  | S |  | S |  | S |
| Cefuroxime |  | R |  | R |  | S |  | S |
| Ceftriaxone | *bla*_TEM-91,180,183_  *bla*_CTX-M-15,88,156,176,193,202_ | R |  | S | *bla*_ACT-5_ | S | *bla*_ACT-5_*_, 14_*  *bla*_OXA-1,224,534_ | S |
| Cefepime |  | S |  | S |  | S |  | S |
| Doripenem |  | R |  | S |  | S |  | S |
| Ertapenem |  |  |  | S |  | S |  | S |
| Imipenem |  | R |  | S |  | S |  | S |
| Meropenem | *bla*_OXA-232_ | R |  | S |  | S | *bla*_OXA-232_ | S |

|  | Urine 25** | | Urine 26** | | Urine 27** | | Urine 28** | |
| --- | --- | --- | --- | --- | --- | --- | --- | --- |
|  | MinION | Culture | MinION | Culture | MinION | Culture | MinION | Culture |
| Bacteria | *E. coli* | *E. coli* | *K. pneumoniae* | *K. pneumoniae* | *E. coli* | *E. coli* | *E. coli* | *E. coli* |
| Ampicillin | *bla*_TEM-1B_ | R |  | R |  | R |  | R |
| Amoxicillin/  Clav |  | R |  | R |  | R |  | I |
| Piperacillin/Taz |  | I |  | S |  | R |  | S |
| Cefazolin |  | R |  | R |  | R |  | R |
| Cefuroxime |  | R |  | R |  | R |  | R |
| Ceftriaxone | *bla*_TEM-105,213_  *bla*_CTX-M-42,55,88,188_  *bla*_OXA-10,50_ | R | *bla*_OXA-1,320,534_  *bla*_CTX-M-15,117,176,197_  *bla*_TEM-1B,70,166,182_ | R | *bla*_ACT-5_  *bla*_CMY-2,4,6,22,62,69,138,146_  *bla*_CTX-M-55,172,173,176,209_  *bla*_TEM-30,57,68,105,158,172,176_  *bla*_VEB-1,3,4,5,6,8_ | R | *bla*_TEM-198_ | R |
| Cefepime |  | R |  | S |  | R |  | R |
| Doripenem |  | S |  | S |  | S |  | S |
| Ertapenem |  | S |  | S |  | S |  | S |
| Imipenem |  | S |  | S |  | S |  | S |
| Meropenem | *bla*_IMP-14,48_ | S |  | S |  | S |  | S |

|  | Urine 29** | | Urine 30** | | Urine 31** | | Urine 32** | |
| --- | --- | --- | --- | --- | --- | --- | --- | --- |
|  | MinION | Culture | MinION | Culture | MinION | Culture | MinION | Culture |
| Bacteria | *E. coli* | *E. coli/*  *Citrobacter koseri* | *E. coli* | *E. coli/*  *Pseudomonas aeruginosa* | *E. coli* | *E. coli* | *Enterobacter cloacae* strain | *Enterobacter cloacae* complex |
| Ampicillin |  | R/- | *bla*_TEM-1B_ | R/- |  | R | *bla*_OXA-1_ | - |
| Amoxicillin/  Clav |  | I/S |  | R/- |  | I |  | R |
| Piperacillin/Taz |  | S/S |  | S/- |  | S |  | S |
| Cefazolin |  | R/- |  | R/- |  | R |  | R |
| Cefuroxime |  | R/S |  | R/- |  | R |  | R |
| Ceftriaxone |  | R/S | *bla*_OXA-1,320_  *bla*_CTX-M-157_  *bla*_TEM-166,188,215_ | R/- | *bla*_CTX-M-27,84,174_  *bla*_TEM-146,217,220_ | R | *bla*_ACT-9_ | R |
| Cefepime |  | R/S |  | R/- |  | S |  | SDD |
| Doripenem |  | S/S |  | S/S |  | S |  | S |
| Ertapenem |  | S/S |  | S/- |  | S |  | S |
| Imipenem |  | S/S |  | S/S |  | S |  | S |
| Meropenem |  | S/S |  | S/S |  | S |  | S |

|  | Urine 33 /** | | Urine 34 | | Urine 35 | | Urine 36* | |
| --- | --- | --- | --- | --- | --- | --- | --- | --- |
|  | MinION | Culture | MinION | Culture | MinION | Culture | MinION | Culture |
| Bacteria | *E. coli* | *E. coli*  2 isolates | *E. coli* | *E. coli* | *K. pneumoniae* | *K. pneumoniae* | *K.*  *pneumoniae* | *K.*  *pneumoniae* |
| Ampicillin |  | R/R |  | R |  | R |  | R |
| Amoxicillin/  Clav |  | S/S |  | I |  | S |  | I |
| Piperacillin/Taz |  | S/S |  | S |  | S |  | S |
| Cefazolin |  | S/R |  | S |  | S |  | S |
| Cefuroxime |  | S/R |  | S |  | S |  | R |
| Ceftriaxone |  | S/R |  | S |  | S |  | S |
| Cefepime |  | S/S |  | S |  | S |  | S |
| Doripenem |  | S/S |  | S |  | S |  | S |
| Ertapenem |  | S/S |  | S |  | S |  | S |
| Imipenem |  | S/S |  | S |  | S | *bla*_SHV-38_ | R |
| Meropenem |  | S/S |  | S |  | S |  | S |

|  | Urine 37* | | Urine 38* | | Urine 39* | | Urine 40* | |
| --- | --- | --- | --- | --- | --- | --- | --- | --- |
|  | MinION | Culture | MinION | Culture | MinION | Culture | MinION | Culture |
| Bacteria | *K. pneumoniae* | *K. pneumoniae* | *K. aerogenes* | *K. aerogenes* | *K. pneumoniae* | *K. pneumoniae* | *E. coli*/*K. pneumoniae* | *E. coli/K. pneumoniae* |
| Ampicillin |  | R |  | - | *bla* _TEM1D_ | R |  | R/R |
| Amoxicillin/  Clav |  | R |  | R |  | R |  | S/R |
| Piperacillin/Taz |  | R |  | S |  | R |  | S/R |
| Cefazolin |  | R |  | R |  | R |  | R/R |
| Cefuroxime |  | R |  | R |  | R |  | R/R |
| Ceftriaxone | *bla*_SHV-26_  *bla*_CTX-M-189_  *bla* _OXA-9_ | R |  | S | *bla*_CTX-M-55,142,180,208-9_  *bla* _OXA-9_  *bla*_TEM-183_ | R | *bla*_ACT-5_  *bla*_SHV11-12_  *bla*_TEM-1B,1C,146,215_  *bla*_CTX-M-19,24,55,127,129,156,192,204_ | R/R |
| Cefepime |  | R |  | S |  | R |  | S/R |
| Doripenem |  | R |  | S |  | R |  | S/I |
| Ertapenem |  | - |  | S |  | - |  | S/- |
| Imipenem |  | R | *bla*_NDM-1_ | I |  | R | *bla*_OXA-232_ | S/R |
| Meropenem | *bla*_OXA-232_ | R |  | S | *bla*_NDM-1,2_ | R |  | S/S |

|  | Urine 41* | | Urine 42* | | Urine 43* | | Urine 44* | |
| --- | --- | --- | --- | --- | --- | --- | --- | --- |
|  | MinION | Culture | MinION | Culture | MinION | Culture | MinION | Culture |
| Bacteria | *K. pneumoniae* | *K.*  *aerogenes* | *K. pneumoniae* | *K. pneumoniae* | - | *K. pneumoniae* | *E. coli* | *E. coli* |
| Ampicillin |  | - | *bla*_OKP-B-3_ | R |  | R | *bla*_OXA-1_ | R |
| Amoxicillin/  Clav |  | R |  | R |  | R |  | R |
| Piperacillin/Taz |  | S |  | S |  | R |  | R |
| Cefazolin |  | R |  | R |  | R |  | R |
| Cefuroxime |  | R |  | R |  | R |  | R |
| Ceftriaxone |  | S | *bla*_DHA-1,27_ | S |  | R | *bla*_TEM-76,206,208,215_  *bla*_LAP-2_ | R |
| Cefepime |  | S |  | S |  | R |  | SDD |
| Doripenem |  | S |  | I |  | R |  | R |
| Ertapenem |  | S |  | S |  | - |  | - |
| Imipenem |  | I |  | S |  | I |  | R |
| Meropenem |  | S |  | S |  | I | *bla*_NDM-1,6_ | R |

|  | Urine 45* | | Urine 46* | | Urine 47* | | Urine 48* | |
| --- | --- | --- | --- | --- | --- | --- | --- | --- |
|  | MinION | Culture | MinION | Culture | MinION | Culture | MinION | Culture |
| Bacteria | *K. pneumoniae* | *K. pneumoniae* | *K. pneumoniae* | *K. pneumoniae* | *E. coli* | *E. coli* | *E. cloacae* complex | *E. cloacae* complex |
| Ampicillin | *bla*_TEM-1B_ | R | *bla*_TEM-1A_ | R | *bla*_TEM-1B_ | R | *bla*_TEM-1B_*, bla*_OXA-1_ | - |
| Amoxicillin/  Clav |  | R |  | R |  | R |  | R |
| Piperacillin/Taz |  | R |  | R |  | R |  | R |
| Cefazolin |  | R |  | R |  | R |  | R |
| Cefuroxime |  | R |  | R |  | R |  | R |
| Ceftriaxone | *bla*_TEM-76,206,208_  *bla*_CTX-M-182_  *bla*_OXA-1_  *bla*_LAP-2_  *bla*_SHV-100_  *bla*_OXA-566_ | R |  | R | *bla*_TEM-34,70,176,200,215,226_  *bla*_ACT-5_  *bla*_OXA-1,31_  *bla*_CTX-M-15,52,79,118,173,183_  *bla*_CMY-2,4,32,107_ | R | *bla*_CTX-M-15,114,117,182,211_  *bla*_ACT-15_  *bla*_OXA-320,392,534_  *bla*_TEM-104,144,196,216_ | R |
| Cefepime |  | S |  | R |  | R |  | R |
| Doripenem |  | R |  | R |  | R |  | R |
| Ertapenem |  |  |  | - |  | - |  | - |
| Imipenem |  | R |  | R |  | R |  | R |
| Meropenem | *bla*_OXA-48_  *bla*_NDM-1_ | R | *bla*_NDM-16_ | R | *bla*_NDM-5,15,17,20,21_ | R | *bla*_NDM-1,3,24_ | R |

|  | Urine 49* | | Urine 50** | | Urine 51** | | Urine 52** | |
| --- | --- | --- | --- | --- | --- | --- | --- | --- |
|  | MinION | Culture | MinION | Culture | MinION | Culture | MinION | Culture |
| Bacteria | *K. pneumoniae* | *K. pneumoniae* | *E. coli* | *E. coli* | *K. pneumoniae* | *K. pneumoniae* | *E. coli* | *E. coli* |
| Ampicillin |  | R | *bla*_OXA-1_ | R | *bla*_SHV-1_ | R |  | R |
| Amoxicillin/  Clav |  | R |  | I |  | I |  | R |
| Piperacillin/Taz |  | R |  | I |  | S |  | S |
| Cefazolin |  | R |  | R |  | R |  | R |
| Cefuroxime |  | R |  | R |  | R |  | R |
| Ceftriaxone | *bla*_CTX-M-15,55,176,189,197,209_  *bla*_OKP-B-18_ | R | *bla*_OXA-31,534_  *bla*_CTX-M-182,184,189,202-3,218_ | R |  | R | *bla*_DHA-1,24_ | R |
| Cefepime |  | R |  | R |  | R |  | S |
| Doripenem |  | R |  | S |  | S |  | S |
| Ertapenem |  | - |  | S |  | S |  | S |
| Imipenem |  | R |  | S |  | S |  | S |
| Meropenem | *bla*_NDM-1,2,6,9,14,16,22,24_ | R |  | S |  | S |  | S |

|  | Urine 53** | | Urine 54** | | Urine 55** | | Urine 56 | |
| --- | --- | --- | --- | --- | --- | --- | --- | --- |
|  | MinION | Culture | MinION | Culture | MinION | Culture | MinION | Culture |
| Bacteria | *E. coli* | *E. coli* | *E. coli* | *E. coli* | *K. pneumoniae* | *K. pneumoniae* | *E. coli* | *E. coli* |
| Ampicillin |  | R |  | R |  | R |  | R |
| Amoxicillin/  Clav |  | R |  | R |  | I |  | S |
| Piperacillin/Taz |  | S |  | I |  | R |  | S |
| Cefazolin |  | R |  | R |  | R |  | S |
| Cefuroxime |  | R |  | R |  | R |  | S |
| Ceftriaxone | *bla*_OXA-534_ | R | *bla*_OXA-224_  *bla*_CTX-M-180,182,184_ | R | *bla*_CTX-M-114,210,216_ | R |  | S |
| Cefepime |  | S |  | R |  | R |  | S |
| Doripenem |  | S |  | S |  | S |  | S |
| Ertapenem |  | S |  | S |  | S |  | S |
| Imipenem |  | S |  | S |  | S |  | S |
| Meropenem |  | S |  | S |  | S |  | S |

|  | Urine 57 | | Urine 58 | | Urine 59 | | Urine 60 | |
| --- | --- | --- | --- | --- | --- | --- | --- | --- |
|  | MinION | Culture | MinION | Culture | MinION | Culture | MinION | Culture |
| Bacteria | - | *K. pneumoniae* | *E. coli* | *E. coli* | *K. pneumoniae* | *K. pneumoniae* | *E. coli* | *E. coli* |
| Ampicillin |  | R |  | R |  | R |  | R |
| Amoxicillin/  Clav |  | S |  | S |  | S |  | S |
| Piperacillin/Taz |  | S |  | S |  | S |  | S |
| Cefazolin |  | S |  | S |  | S |  | S |
| Cefuroxime |  | S |  | S |  | S |  | S |
| Ceftriaxone |  | S | *bla*_TEM-127,144,176_ | S |  | S |  | S |
| Cefepime |  | S |  | S |  | S |  | S |
| Doripenem |  | S |  | S |  | S |  | S |
| Ertapenem |  | S |  | S |  | S |  | S |
| Imipenem |  | S |  | S |  | S |  | S |
| Meropenem |  | S |  | S |  | S |  | S |

S – susceptible, I – intermediate, R – resistant, SDD – susceptible dose dependent

*Carbapenem-resistant Enterobacteriaceae (CRE phenotype, excluding *Proteus mirabilis* with intrinsic resistance to imipenem)

**3^rd^-generation cephalosporin-resistant Enterobacteriaceae (still susceptible to all carbapenems—ESBL/AmpC phenotype)

- For comparison, ampicillinase genes are filled in the table cells next to ampicillin susceptibility results, ESBL/AmpC genes next to ceftriaxone, and carbapenemase genes next to meropenem or other non-susceptible carbapenems.
- Dark gray color highlights the discordances between genotypic (MinION) and phenotypic (antimicrobial susceptibility test) resistance to carbapenems and 3^rd^-generation cephalosporins.
- In case of more than 1 Enterobacteriaceae isolate per sample with different susceptibility, the result with the highest degree of resistance is chosen to represent that sample phenotype.

**Table S2.** Comparison between prescribed antibiotics, antibiotic suggested by Nanopore sequencing, and antibiotics suggested by AST (n=35)

| Urine | Prescribed antibiotics | Antibiotics  suggested by Nanopore | Antibiotics  suggested by standard AST | Coverage of antibiotics  suggested by Nanopore | Coverage of prescribed antibiotics | Strategy suggested by Nanopore | Comparing Nanopore to prescribed  antibiotics* |
| --- | --- | --- | --- | --- | --- | --- | --- |
| 1 | ceftazidime | colistin | colistin | optimal | too narrow | escalation | better |
| 3 | ceftazidime | carbapenems | colistin | too narrow | too narrow | escalation | similar |
| 6 | ceftazidime | carbapenems | carbapenems | optimal | too narrow | escalation | better |
| 8 | ceftazidime | colistin | colistin | optimal | too narrow | escalation | better |
| 9 | cefixime | ceftriaxone | ceftriaxone | optimal | optimal | continue | similar |
| 11 | ceftriaxone | carbapenems | ceftriaxone | too broad | optimal | escalation | worse |
| 15 | ceftriaxone | colistin | colistin | optimal | too narrow | escalation | better |
| 16 | ceftriaxone | colistin | colistin | optimal | too narrow | escalation | better |
| 17 | colistin | colistin | colistin | optimal | optimal | continue | similar |
| 18 | ceftriaxone | colistin | colistin | optimal | too narrow | escalation | better |
| 19 | ceftazidime | ceftriaxone | colistin | too narrow | too narrow | de-escalation | similar |
| 20 | ceftazidime | colistin | colistin | optimal | too narrow | escalation | better |
| 22 | ceftriaxone | ceftriaxone | ceftriaxone | optimal | optimal | continue | similar |
| 23 | ceftazidime | carbapenems | ceftriaxone | too broad | too broad | escalation | worse |
| 24 | cefixime | colistin | ceftriaxone | too broad | optimal | escalation | worse |
| 25 | ceftazidime | carbapenems | carbapenems | optimal | too narrow | escalation | better |
| 26 | ceftazidime | carbapenems | carbapenems | optimal | too narrow | escalation | better |
| 27 | ceftazidime | carbapenems | carbapenems | optimal | too narrow | escalation | better |
| 29 | ceftazidime | ceftriaxone | carbapenems | too narrow | too narrow | de-escalation | similar |
| 32 | ceftriaxone | carbapenems | carbapenems | optimal | too narrow | escalation | better |
| 33 | ceftriaxone | carbapenems | carbapenems | optimal | too narrow | escalation | better |
| 34 | cefixime | carbapenems | ceftriaxone | too broad | optimal | escalation | worse |
| 35 | ceftriaxone | ceftriaxone | ceftriaxone | optimal | optimal | continue | similar |
| 36 | ceftriaxone | ceftriaxone | ceftriaxone | optimal | optimal | continue | similar |
| 39 | meropenem | colistin | colistin | optimal | too narrow | escalation | better |
| 40 | piperacillin/  tazobactam | colistin | colistin | optimal | too narrow | escalation | better |
| 42 | ceftazidime | carbapenems | ceftriaxone | too broad | too broad | escalation | worse |
| 45 | cefixime | carbapenems | colistin | too narrow | too narrow | escalation | similar |
| 51 | ceftazidime | ceftriaxone | carbapenems | too narrow | too narrow | de-escalation | similar |
| 52 | ceftazidime | ceftriaxone | carbapenems | too narrow | too narrow | de-escalation | similar |
| 53 | ceftriaxone | ceftriaxone | carbapenems | too narrow | too narrow | continue | similar |
| 54 | meropenem | carbapenems | carbapenems | optimal | optimal | continue | similar |
| 57 | ceftriaxone | ceftriaxone | ceftriaxone | optimal | optimal | continue | similar |
| 59 | ceftriaxone | ceftriaxone | ceftriaxone | optimal | optimal | continue | similar |
| 60 | piperacillin/  tazobactam | ceftriaxone | ceftriaxone | optimal | too broad | de-escalation | better |

AST – antimicrobial susceptibility test

*Criteria for the comparison results:

- Better
  - An antibiotic suggested by Nanopore was optimal, while a prescribed antibiotic was not optimal (too broad or too narrow).
  - Both were too broad, but a prescribed antibiotic was even broader than an antibiotic suggested by Nanopore.
  - An antibiotic suggested by Nanopore was too broad, while a prescribed antibiotic was too narrow. (Not found in this study)
- Similar
  - An antibiotic suggested by Nanopore was the same or similar in spectrum to a prescribed antibiotic.
  - Both antibiotic suggested by Nanopore and prescribed antibiotic were too narrow regardless of whether which one was narrower.
- Worse
  - An antibiotic suggested by Nanopore was not optimal (too broad or to narrow), while a prescribed antibiotic was optimal.
  - Both were too broad, but an antibiotic suggested by Nanopore was even broader than a prescribed antibiotic.
  - An antibiotic suggested by Nanopore was too narrow, while a prescribed antibiotic was too broad. (Not found in this study)
